# On electrochemistry of metal–organic framework Zn_2_(EDTA)(H_2_O)†

**DOI:** 10.1039/d3ra00040k

**Published:** 2023-02-07

**Authors:** Alena A. Starodubtseva, Yaroslav S. Zhigalenok, Kairgali M. Maldybaev, Alina K. Galeyeva, Ivan A. Trussov, Andrey P. Kurbatov

**Affiliations:** a Center of Phisico-Chemical Methods of Research and Analysis, Al-Farabi Kazakh National University Almaty 010000 Kazakhstan ivan.trussov@hotmail.com; b Skoltech Center for Electrochemical Energy Storage, Skolkovo Institute of Science and Technology Moscow 121205 Russian Federation

## Abstract

Metal–organic compounds (MOFs) are a class of substances composed of metal ions or clusters coordinated to organic ligands to form one-, two-, or three-dimensional structures. Due to their high porosity, excellent adsorption and catalytic activity, as well as the possibility of simultaneous implementation of various charge accumulation mechanisms, they can be used as electrode materials for metal-ion batteries. However, a significant disadvantage is that most MOFs have a low electrical conductivity, and the production of conductive MOFs is a costly, time-consuming and technically difficult process. In this work, we developed a method for synthesizing the Zn_2_(EDTA)(H_2_O) MOF composite and studied the possibility of using it as an anode material for sodium-ion batteries based on aqueous electrolytes. The structure and morphology of the compound was studied using XRD, IR, TGA and SEM. Using cyclic voltammetry, the electrochemical characteristics of the organometallic framework in alkaline electrolytes 1, 10 M NaOH, as well as in saturated aqueous electrolyte NaClO_4_, were evaluated. It has been established that the studied compound does not give a satisfactory electrochemical response in aqueous electrolytes (both in alkaline and neutral media) due to the strong degradation of the electrode material, which is associated with the high solubility of this MOF representative. Cyclic voltammetric studies showed the presence of two redox processes due to the release of metallic zinc from an electrolyte solution, where two forms of zinc exist in equilibrium (the ZnEDTA complex and the free zinc cation). Therefore, we concluded, it is not possible to use this material as an anode for water-based sodium-ion batteries in contrary to a published research study.

## Abstract 2

Zn_2_(EDTA)(H_2_O) irreversibly decomposes in both neutral (sat. NaClO_4_) and alkali (NaOH) aqueous electrolytes with formation of various Zn complexes.

## Graphical abstract

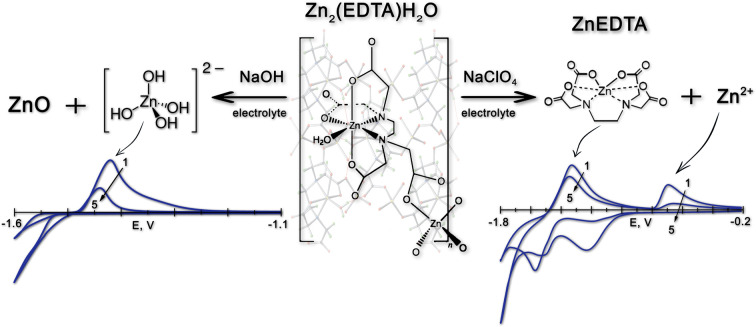

## 1 Introduction

Lithium-ion batteries (LIB) are now ubiquitous and can be found in almost any portable electronics. They have a high energy density, high operating voltage, low self-discharge and a large number of charge–discharge cycles.^1,2^ However, the limited resources of lithium, the location of deposits in politically unstable regions and the unsafe operation of lithium-ion batteries, associated with the risk of leakage, create prerequisites for the search and study of new battery systems.^3,4^ Sodium-ion batteries (SIBs) are increasingly attracting the attention of scientists due to the high abundance of sodium in the earth's crust, combined with their low cost. In addition, the sodium ion has a lower cation solvation energy in polar solvents and higher mobility in electrolytes compared to lithium, promising improved cell performance.^3^ The main problem faced in the development of Na-ion batteries is the search for anode materials with high specific capacity and stability to implement a reversible sodium intercalation/deintercalation process, since classical lithium-ion battery anodes are not suitable for sodium.^5^ One of the promising directions for solving this problem is the use of metal–organic frameworks (MOFs) as anodes. MOFs are a class of compounds consisting of metal ions or clusters coordinated to organic ligands to form one-, two-, or three-dimensional structures with formation of complex 3D tunnels for ion migration.^6^ Such structural properties already stated a large range of MOFs as excellent adsorbers, however these also open a way for alkali ion intercalation therefore opening prospects for battery applications especially for sodium lacking sustainable anode option. Since the synthesis of the first metal–organic framework by Yaghi and Li in the 1990s,^7,8^ more than 20 000 representatives with controlled size, shape and characteristics have been obtained. Representatives of this class of substances are able to form structures with bulky cavities in the crystal lattice, which makes possible the reversible intercalation of sodium ions, including in aqueous or organic electrolytes.^9,10^ Previous studies have shown that during the intercalation/deintercalation of sodium ions in MOF, a reversible RedOx process involving transition metal ions is possible. However, many MOFs have low electrical conductivity, which requires the formation of a conductive coating on the surface of the particles.^11–13^ Moreover, MOFs often undergo irreversible structural degradation during cycling hindering their practical use. The first work on the use of MOF as an anode material for metal-ion batteries was done with MOF-177 (Zn_4_O(BTB)_2_ × (DEF)_*m*_ × (H_2_O)_*n*_).^14^ Chen *et al.* found that there was a rapid decrease in the capacity of MOF-177 from 400 mA h g^−1^ in the first cycle to 105 mA h g^−1^ in the second cycle due to the destruction of the metal–organic framework as a result of irreversible reduction zinc in the structure to the metallic state. Similar problems were identified by Liu *et al.* when investigating (Mn(TFBDC)(4,4-BPY)(H_2_O)_2_) or Mn-LCP as an electrode material.^15^ Subsequently, Saravanan *et al.* showed that the reversibility of the process during cycling can be improved by replacing the organic ligand. They successfully used Zn_3_(HCOO)_6_, Co_3_(HCOO)_6_, and Zn_1.5_Co_1.5_(HCOO)_6_ as anode materials. Zn_3_(HCOO)_6_ showed the best initial capacity of 693 mA h g^−1^, subsequently decreasing to 560 mA h g^−1^ after 60 cycles. Co_3_(HCOO)_6_ MOF and Zn_1.5_Co_1.5_(HCOO)_6_ MOF showed 410 mA h g^−1^ and 510 mA h g^−1^ reversible capacity, respectively, after 60 cycles. These MOFs show good long cycle capability, high Faraday efficiency, as the zinc and cobalt formate frameworks react reversibly with the lithium ion to form lithium formate. Lin *et al.* then developed Zn-MOF (BMOF), which contains phenyl and amine functional groups.^16^ This material has shown excellent chemical and thermal stability. Zn-MOF was used as the active anode material, which showed a capacity of 190 mA h g^−1^ after 200 cycles.

However, all of these studies have been done using organic electrolytes, which are toxic, volatile, and expensive.^17^ To make renewable energy production cheaper and more environmentally friendly, battery systems based on aqueous electrolytes are being researched. Aqueous electrolytes are considered promising candidates due to their safety, low cost, high ionic conductivity.^18^ For many years, aqueous electrolytes have been considered unsuitable for use in batteries due to the limited electrochemical stability window of water, which ranges from 1.23 V (thermodynamic stability limit) to 1.5 V in dilute electrolyte solutions.^19^ One way to solve this problem is the use of highly concentrated electrolytes, since there is a shift in the overpotential of oxygen and hydrogen evolution during water decomposition, which, in turn, increases the operating voltage of battery systems. In such electrolytes, an increased cation concentration provides a shift in the equilibrium potential of the anode reaction material towards more positive values, which makes it possible to minimize the contribution of the parasitic reaction of hydrogen evolution.^17^ In metal–organic frameworks, the M–C and M–H bonds in transition metal compounds have a low polarity, so they usually do not interact with water. This makes MOFs promising electrode materials for batteries based on aqueous electrolytes.

Gelabert *et al.* had synthesized Zn_2_(EDTA)H_2_O having a complex 3D MOF framework.^20^ Later Patel *et al.* conducted electrochemical studies of this material in 0.1 M KOH solution.^21^ In their study, the Zn_2_(EDTA)H_2_O-based electrode showed a high specific capacity of 243.2 mA h g^−1^ at a current density of 1 A g^−1^ and stability for 8000 cycles without significant capacity loss. At the same time, the authors claim that the presence of pronounced cathode and anode peaks in CV is explained by the reversible surface reaction Zn^2+^ ⇌ Zn^+^. According to the literature data, the free zinc ion in the +1 oxidation state is extremely unstable and occurs only in the form of a dimeric structure with a Zn–Zn bond,^22^ nevertheless, the explanation of the stability of the Zn^+^ particle and the directly detailed mechanism of the reversible RedOx process were not presented by Patel *et al.* In addition, according to the galvanostatic cycling curves, the electrochemical activity of this material in an alkaline electrolyte is manifested in the potential range from −0.10 V to 0.25 V (*vs.* Hg_2_Cl_2_/Cl^−^). In aqueous solutions of electrolytes, the potential of the Zn^2+^/Zn pair is close to the potential of the onset of hydrogen evolution from water (*E*_0_ = −0.76 V, pH < 7).^23^ In the structure of Zn_2_(EDTA)H_2_O, the presence of ligands around zinc, binding it in the crystal lattice, will probably contribute to an even greater shift in its reduction potential to a more negative region relative to the free zinc ion.^24^ Therefore, it is expected that the electrochemical activity of the material should manifest itself in the region of negative potentials.

In this paper, we question some of the statements made and the subsequent conclusions drawn in the work of Patel *et al.*,^21^ namely the stability and long-term cycling of Zn_2_(EDTA)H_2_O in an aqueous electrolytes. We show a different mechanism of the electrochemical process in a composite based on Zn_2_(EDTA)H_2_O in aqueous electrolytes. Here we discuss the effect of media on the stability of the Zn_2_(EDTA)H_2_O metal–organic framework during electrochemical studies, as well as the possibility of using Zn_2_(EDTA)H_2_O as an anode material for sodium-ion batteries based on highly concentrated aqueous sodium electrolytes.

## 2 Experimental

To prepare Zn_2_(EDTA)H_2_O MOF, zinc chloride ZnCl_2_ (0.085 mol, Aldrich, 98%) was dissolved in distilled water (20 ml). After that Na_2_H_2_EDTA·2H_2_O powder (0.085 mol , Aldrich, 99%) was added to the salt solution with constant stirring. Then, a solution of 1 M potassium hydroxide KOH (Aldrich, 99%) was added drop-wise until the mixture was completely dissolved and a pH of 3.0–3.4 was reached. After measuring the pH, the solution was placed in an autoclave with a 100 ml Teflon liner. The autoclave was placed in an oven for 24 hours at a temperature of 180 °C. After cooling the autoclave to room temperature, the obtained crystals were separated from the solution by repeated decantation-washing, and dried at a temperature of 120 °C for 4 hours in an oven.

Powder X-ray diffraction in the Bragg–Brentano geometry was carried out on a DRON-7M diffractometer (Burevestnik) with a copper X-ray source (*λ*_CuKα1_ = 1.54056 Å, *λ*_CuKα2_ = 1.54439 Å). Structure refinement by the Rietveld method was carried out using the TOPAS software package with the TOPAS-Academic script library.^25^ Scanning electron microscopy (SEM) on a Quanta 200i 3D (FEITM) instrument was used to study the microstructure and surface morphology. The particle size of the synthesized materials was determined using a Partica LA-960 (HORIBA) laser analyzer in an aqueous medium. Thermogravimetric analysis (TGA) was performed on a F3 STA-449 apparatus (Netzsch, Germany) combined with a mass spectrometer QMS 403 D Aeolos (Netzsch, Germany) in a nitrogen atmosphere with heating at a rate of 5 °C min^−1^ to 600 °C.

For the manufacture of the electrode, the obtained material was preliminary ground using a planetary mill Pulverisette 6 (FRITSCH) in a zirconium oxide mortar at 400 rpm for 4 hours. An anode mass in the form of a paste was obtained by grinding Zn_2_(EDTA)H_2_O, TIMCAL SUPER C45 conductive additive, polyvinylidene fluoride (PVDF) in *N*-methylpyrrolidone (3 wt%) in an agate mortar in a ratio (70 : 20 : 10). The resulting paste was applied in a thin layer using a Doctor Blade (100 μm) onto graphite foil. The resulting electrode was dried at a temperature of 70 °C for an hour in an oven and then placed in a vacuum oven for 24 hours at a temperature of 120 °C.

Electrochemical measurements were performed by cyclic voltammetry using an Autolab PGSTAT 302N potentiostat-galvanostat. A three-electrode cell with NaOH (1 M, 10 M) aqueous electrolytes and a saturated NaClO_4_ solution was used for the studies at a sweep rate of 10 mV s^−1^. Such a sweep rate was selected since at lower scanning rates the media induced ZnMOF dissolution becomes overwhelming due to longer time of exposure to the electrolyte, therefore, the results cannot be interpreted reliably. The higher speed of scanning affects the CV curve too strongly due to catching only very surface of the electrode already covered with decomposition products. A silver chloride electrode (3.5 M KCl) served as a reference electrode, relative to which all potential values are given, a carbon cloth was used as an auxiliary electrode.

## 3 Results and discussion

Synthesis of the compound Zn_2_(EDTA)H_2_O (Zn_2_C_10_N_2_O_8_H_12_[H_2_O]) by the hydrothermal method was carried out according to the method published by Patel *et al.*^21^ The whole range of experiments is described in the Table S1† in ESI. After the process in an autoclave, no formation of the desired compound was detected, the solution turned yellow and the formation of gaseous products was observed, indicating the decomposition of the reagents. Preliminary degassing and temperature reduction also did not lead to the formation of a solid phase. It was suggested that the amino group of EDTA can interact with the nitrate ion of the zinc salt used leading to the oxidation and decomposition of the organic ligand. To test this hypothesis, zinc nitrate was replaced by chloride, similar to the synthesis of Zn_2_(EDTA)H_2_O by Gelabert *et al.*^20^ During the synthesis with zinc chloride under the same conditions as initially according to Patel *et al.*, no visible changes in the solution were observed, but the solid phase also did not form probably due to the incompleteness of the process. In view of this, the synthesis was carried out at a temperature of 200 °C for 7 days according to the procedure described in the work by Gelabert *et al.*^20^ At the end of the process, the formation of crystals of the desired compound was confirmed on the walls and bottom of the reactor, while the appearance of gaseous products was not observed. Since Gelabert *et al.* grew a single crystal to study the structure of the resulting substance using X-ray diffraction analysis, they sought to obtain particles of the largest size (1–5 mm). However, for the purposes of our work, an anode material with the smallest particle size was required to study its electrochemical properties. For this reason, it was decided to shorten the synthesis time in order to obtain a finer crystalline fraction of the material. We carried out syntheses with different duration from 18 to 96 hours. It was found that after 72 hours the yield of the product is 63% by weight with an average particle size of ∼45 μm and changes slightly with increasing exposure time. Also, in each experiment, the appearance of an additional resinous organic phase and the color of the solution in yellow were observed, which suggests the decomposition of the reagents or the formation of a soluble by-product. To study the patterns of formation of the target product, the influence of pH and temperature on the course of synthesis was studied. Varying the pH in the range of 1.6–5.4 units did not give a significant improvement in the reaction yield.

To study the influence of temperature, the synthesis was carried out at various regimes (100–220 °C). It was found that the formation of Zn_2_(EDTA)H_2_O crystals occurs in the temperature range from 170 to 200 °C. At 180 °C and below, the appearance of a resinous organic phase on the walls and bottom of the vessel and a change in the color of the solution are not observed indicating the absence of a side reaction. It was found that the maximum mass yield of 71% is achieved at a temperature of 180 °C and a more finely crystalline fraction with an average particle size of ∼42 μm is formed. In addition, the influence of the concentration of reagents on the synthesis process was studied. A 2× increase in concentration does not affect the yield of the product, while a 3× increase leads to the formation of a resinous organic phase.

**Fig. 1:**
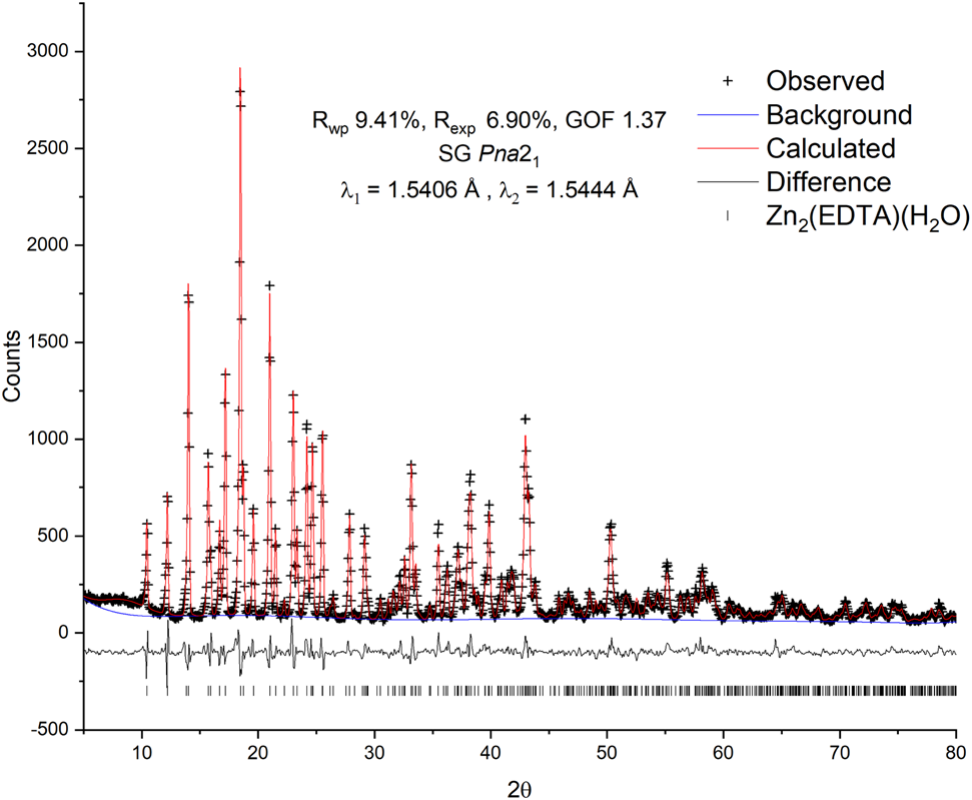
Observed, calculated and difference plots of the Rietveld structure refinement of the Zn_2_(EDTA)H_2_O sample.

To confirm the structure of the obtained substance, X-ray diffraction analysis was carried out, followed by refinement of the structure by the Rietveld method^26^ (Fig. 1). The diffraction pattern of the sample was successfully indexed in the orthorhombic space group *Pna*2_1_ with unit cell parameters: *a* = 12.7995(13) Å, *b* = 11.2517(12) Å, *c* = 9.4622(10) Å, Vol. = 1362.7(3) Å^3^. The obtained values are in good agreement with the values previously obtained by Gelabert *et al.* in the single crystal X-ray diffraction study.^20^ Additional unidentified peaks, as well as a pronounced halo in the range of 15–30° 2*θ*, were not found, which suggests the high purity of the obtained sample, as well as the absence of significant amounts of amorphous impurities. A small halo centered at ∼9° is likely related to the contribution of the instrument.

For refinement in this work the model of Gelabert *et al.*^20^ was used as the starting point. To correct the signal with reasonable reliability, hydrogen atoms were used in the refinement to account for the electron density in the volume of the unit cell. The result of the calculation confirmed the conformity of the structure of the obtained sample with the previously published. The refinement converged with a high degree of statistical agreement (GOF = 1.37) with the observed data (Fig. 1), while the detected slight deviations in the shape of the peaks can be attributed to the instrumental contribution. Thus, no excess electron densities indicating the presence of additional water molecules in the structure were found. There are two Zn crystallographic positions in the structure: in the tetrahedral (Zn2 – ZnO_4_) and in the octahedral (Zn1 – ZnO_4_N_2_) environment. Due to the delocalization of the charge of the carboxylate anion COO–, the average lengths of Zn–O bonds for both tetrahedral (1.27(8) Å) and octahedral (1.29(11) Å) zinc are equivalent and, accordingly, close. In this case, one water molecule is a part of the Zn1 octahedral environment and is probably quite strongly bound to it.

Additionally, to identify the obtained sample, studies were carried out using IR spectroscopy (Fig. S1†). The spectrum shows an absorption band with a maximum at a wave number of 3301 cm^−1^ in the region of O–H^27–29^ stretching vibrations, which probably refers to a crystallization water (Table 1). This is also evidenced by the peak at 3026 cm^−1^, which falls into the region of manifestation of stretching vibrations of bound –O–H intracomplex compounds.^29^ The bands at wave numbers 740 cm^−1^ and 728 cm^−1^ can also be attributed to the pendulum vibration of water *ρ*(H_2_O).^28^ The spectrum contains four bands of the asymmetric stretching vibration of the carboxylate ion *ν*_as_ COO^−^ at wave numbers 1623, 1612, 1601, 1584 cm^−1^,^27,28,30^ which testifies to the unevenness of the bonds in them. This is probably due to the specificity of the spatial environment of 4 and 6 coordinated zinc,^20^ which gives rise to different energy states of the carboxyl group. It is known that the stretching vibration band for the coordinated COO group lies within 1650–1590 cm^−1^. For the COO group bound to the zinc ion, absorption occurs in the range 1610–1590 cm^−1^, while the stretching vibration band of the free ionized COO^−^ group usually appears at 1630–1575 cm^−1^.^28^ Therefore, the bands of the coordinated and free groups COO is difficult to separate if a zinc salt is used to form the complex. In the spectrum, the most intense bands of the asymmetric stretching vibration of the carboxylate ion *ν*_as_ COO^−^ at 1612, 1584 cm^−1^ are shifted beyond the 1610–1590 cm^−1^ range of the COO group coordinated by zinc. Symmetrical stretching vibrations of the carboxylate ion *ν*_s_ COO^−^ appear at 1419, 1401, 1388 cm^−1^.^27,28,30^ The frequency difference *Δ*_as−s_ for the most intense *ν*_as_ COO^−^ bands are: *Δ*_as−s_ = 193, 211, 224, 165, 183, 196 cm^−1^ (1612–1419 = 193 cm^−1^; 1612–1401 = 211 cm^−1^; 1612–1388 = 224 cm^−1^; 1584–1419 = 165 cm^−1^; 1584–1401 = 183 cm^−1^; 1584–1388 = 196 cm^−1^). The frequency difference for the most intense bands *ν*_as_ COO^−^ in Na_2_H_2_EDTA·2H_2_O is 232 cm^−1^. Therefore, there is a noticeable decrease in the value of *Δ*_as−s_, which is natural for the coordination of a polydentate ligand.

**Table 1:** Characteristic vibrational frequencies of bonds in the metal–organic compound ZnMOF

Groups and types of vibrations	Wave number, cm^−1^
**Shortwave region of the spectrum**	
Stretching vibrations –O–H	3301
Stretching vibrations of bound –O–H intracomplex compounds	3026
Stretching vibrations –C–H	2985, 2948, 2929
Deformation vibrations *δ*CH_2_	1450, 1329, 1311
Stretching vibrations –C–N–	1107, 1078
Stretching vibrations –C–C–	936
Pendulum oscillation of water *ρ*(H_2_O)	740, 728
Asymmetric stretching vibrations carboxylate ion *ν*_as_ COO^−^	1623, 1612, 1601, 1584

**Long-wavelength region of the spectrum**	
Stretching vibrations (MN) characteristic of EDTA complexes with Zn(ii)	500–400
Stretching vibrations of ZnO_4_ polyhedra	500–400
Stretch vibration *ν* (Zn–N)	410
Stretching vibrations of ZnO_6_ polyhedra	322

Stretching vibrations of Zn–N bonds^28,29^ and stretching vibrations of polyhedra ZnO_4_, ZnN_2_O_4_ (ref. 31) are manifested in the long-wavelength region of the spectrum. Stretching vibrations of ZnO_4_ polyhedra also appear in the spectrum in the range of 500–400 cm^−1^,^30^ where the calculated frequency of the characteristic band for ZnO_4_ polyhedra is 485 cm^−1^. In the specified range of our spectrum, two bands were recorded with maxima at wave numbers 472 cm^−1^ and 410 cm^−1^. The band at the wave number 472 cm^−1^ is close in position to the calculated value of the Zn–O bonds 485 cm^−1^ in ZnO_4_ polyhedra. Whereas the band at 410 cm^−1^ corresponds to the stretching vibration *ν* (Zn–N), in a similar way as in the compound [Zn(NH_3_)_4_]I_2_.^28^ The band at the wave number 322 cm^−1^ falls into the region of manifestation of ZnO_6_ polyhedra.^31^ Thus, the data of IR spectroscopy are in good agreement with the data of X-ray phase analysis analyzed above, where the presence of two zinc ions in different coordination (octahedral and tetrahedral) was shown in the crystal structure.

**Fig. 2:**
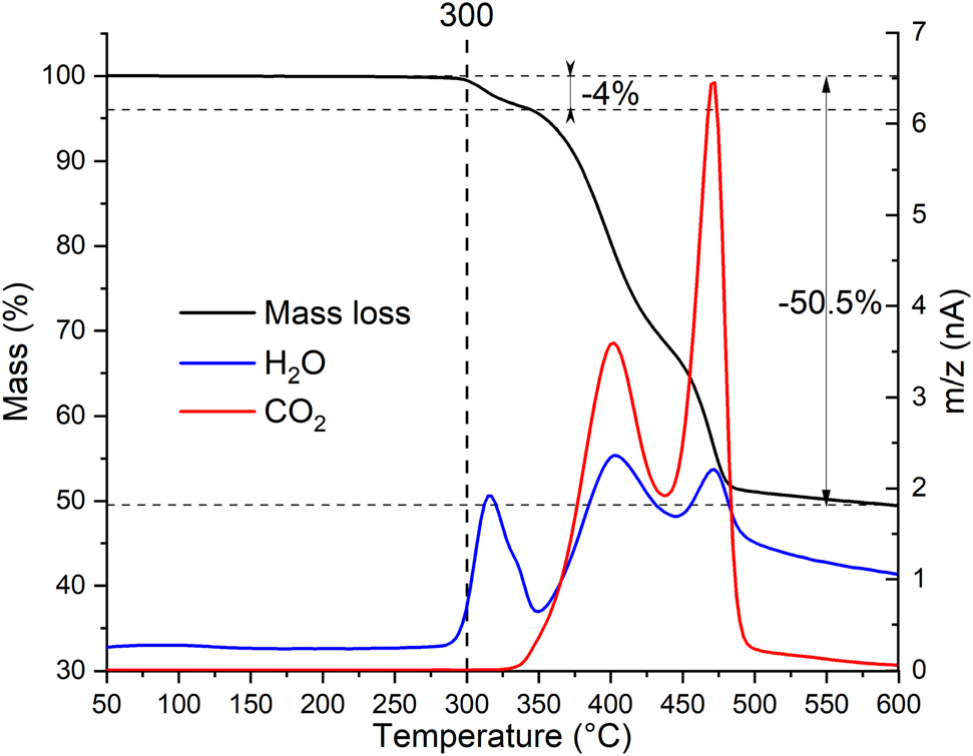
Thermogravimetric analysis with simultaneous detection of evolved gases by mass-spectrometry of Zn_2_(EDTA)H_2_O sample.

To determine the thermal stability window of the Zn_2_(EDTA)H_2_O sample, a thermogravimetric analysis was performed. It was shown that the sample has no mass loss until 300 °C. Then in three steps by 600 °C it loses 50.5% of mass (Fig. 2). This mass loss is obviously associated with structural decomposition of the sample. According to the MS curve an elimination of water begins at 300 °C. The first step in the mass loss (4%) matches with the amount of structural water present in the compound (1 molecule of water corresponding to 4.11% of mass). The structural water loss ends at 350 °C with the beginning of the decomposition of the organic part. The corresponding CO_2_ MS curve shows two peaks corresponding to two remaining steps of the ZnMOF decomposition. The main decomposition process ends by 500 °C. However, we have to consider that in this experiment the material did not undergo the complete decomposition, otherwise one would expect the final mass loss of 62.8% corresponding to ZnO remaining. Considering the observed results, it can be concluded that the material is stable up to 300 °C. These results contradict the data obtained by Patel *et al.*, where it is shown that the material begins to decompose already at a temperature of 150 °C. Most likely the sample in their work had a very significant amount of adsorbed water causing such a significant mass loss at the initial stage.^21^

**Fig. 3:**
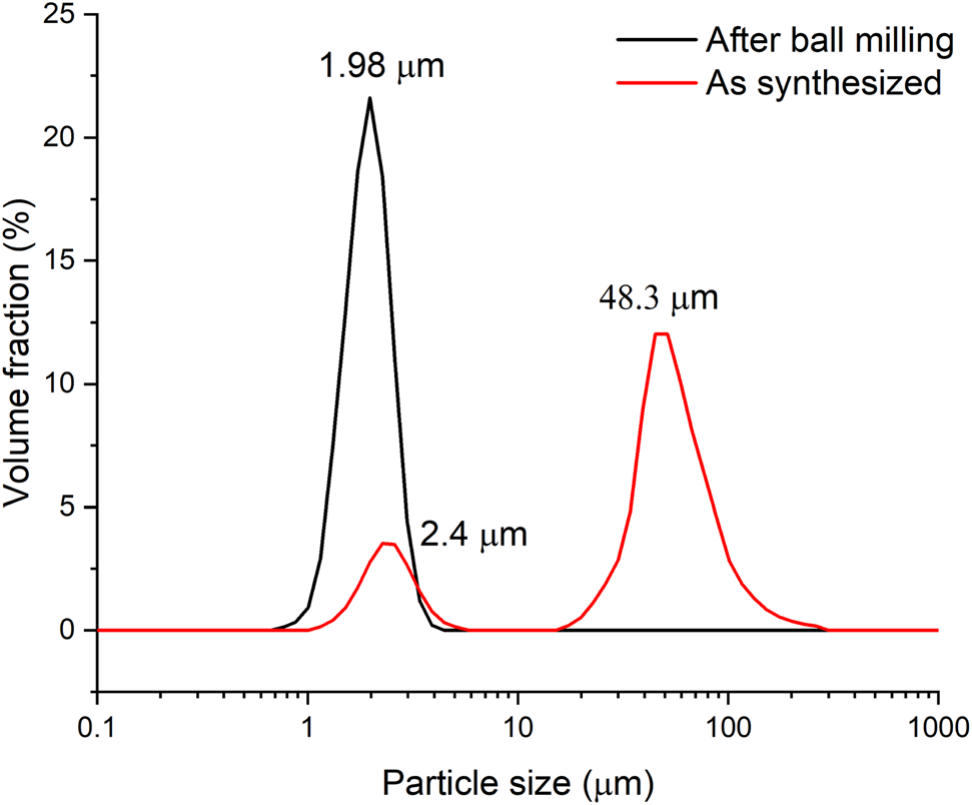
Particle size distribution of Zn_2_(EDTA)H_2_O sample material before and after grinding.

To conduct electrochemical studies, the sample was preliminary ball-milled to reduce the particle size and, accordingly, to reduce the cation and electron transfer path length in the solid phase and subsequently to provide a more complete charge of the material. To test the grinding efficiency, the particle size distribution was determined by the laser dispersion analysis (LDA). Fig. 3 shows that prior to milling, the Zn_2_(EDTA)(H_2_O) sample consists of two populations of microsized particles with median sizes of 2.4 and 48.3 μm. In the process of grinding, the size and polydispersity of the particles of the obtained material decreased, as a result of which their average median size became ∼2 μm.

**Fig. 4:**
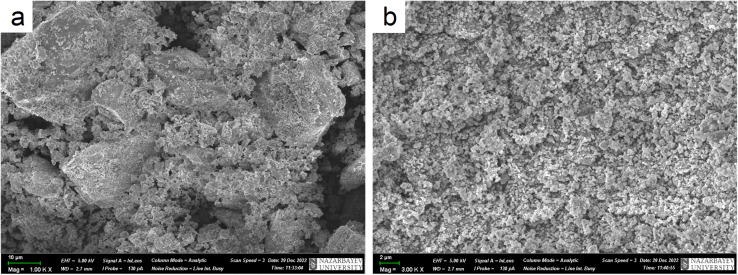
SEM images of Zn_2_(EDTA)H_2_O sample (a) before ball-milling; (b) after ball-milling.

To study the morphology of Zn_2_(EDTA)H_2_O before and after ball-milling samples were studied by scanning electron microscopy (SEM) (Fig. 4). The SEM images show that pristine Zn_2_(EDTA)H_2_O has a wide variety of crystalline particles ranging in sizes from 50 to 1 μm and less. After ball-milling the particles decreased in sizes down to 100 nm (Fig. S3†). These results agree well with LDA measurements considering that light dispersion methods are sensitive to agglomerates formed of hundreds nm particles.

**Fig. 5:**
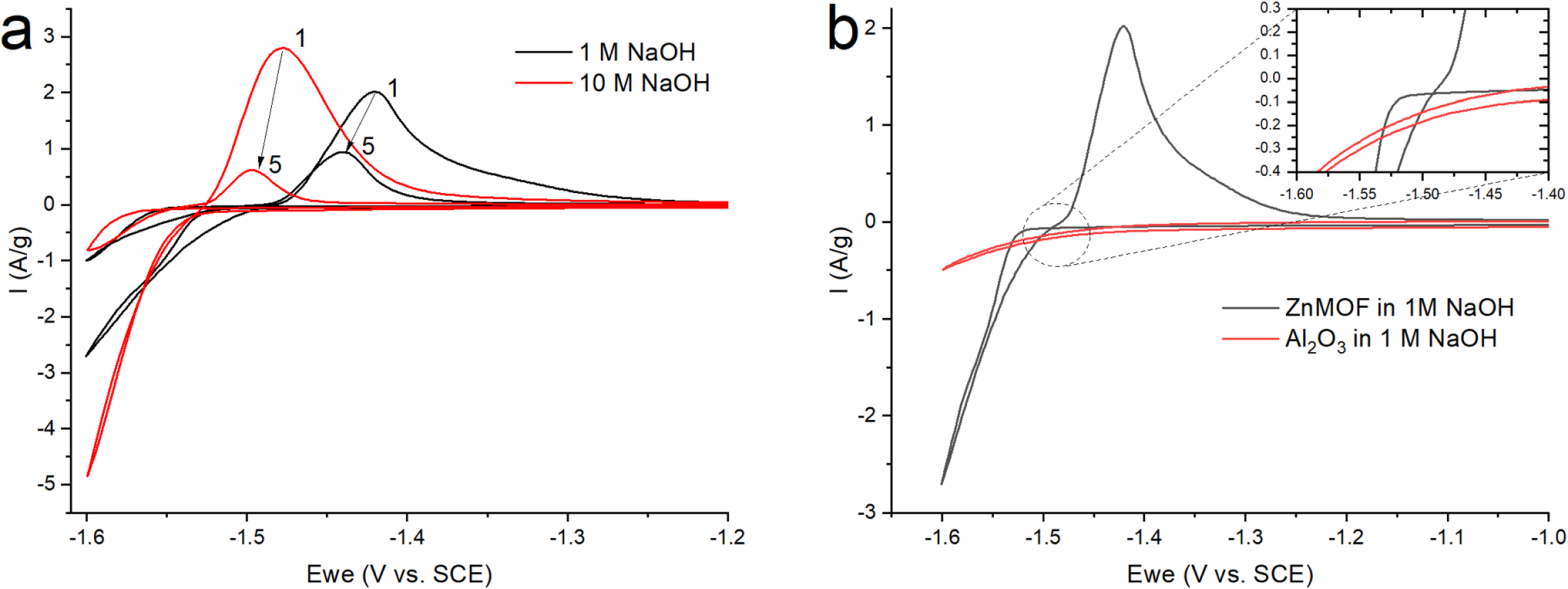
CV (a) ZnMOF in NaOH at various concentrations (b) ZnMOF and Al_2_O_3_ in 1 M NaOH at a sweep rate of 10 mV s^−1^.

After grinding the material in a ball mill, an electrode was made according to the standard method described in the experimental part. Since early work with this material was carried out in an alkaline electrolyte,^21^ to study the electrochemical process of the resulting ZnMOF composite in an alkaline solution, cyclic voltammetry was carried out in 1 and 10 M NaOH solutions (Fig. 5a). The obtained cyclic voltammograms show that a process occurs in the cathode region, probably accompanied by a decrease in the polarizability of the electrode. A change in the polarizability of an electrode may be due to a change in the surface area of the electrochemical process or its nature. This manifests itself in the form of a hysteresis loop, when in the reverse course of the potential sweep, each current value corresponds to a lower overpotential value, as a result of which the polarization curve is shifted to a more positive side relative to the forward course, despite the fact that the cathodic process takes place in this case. This is in a good agreement with the much higher polarization resistance in the initial section of the cathodic polarization curve of the forward stroke compared to the reverse stroke (22.2 kOhm *vs.* 8 kOhm). The calculation of the amount of electricity in the cathode and anode regions on the CV indicates that only 83% of the amount of electricity consumed in the cathode part corresponding to the anode process. This can be explained by the process of hydrogen evolution, which corresponds to the potentials in this region. By itself, the course of the cathode curve in the forward direction corresponds to the exponent of the possible release of hydrogen. To test this assumption, we fabricated electrodes without an active mass of MOFs with an inert filler in the form of aluminum oxide. In this case, aluminum oxide is the ballast and occupies the same proportion in the electrode as the MOF working material. On such an electrode, the only process in an alkaline electrolyte in this range of potentials will be the evolution of hydrogen (Fig. 5b). Therefore, in the ideal case, we can evaluate the contribution of the parallel process of hydrogen evolution on the carbon surface of the MOF composite. The amount of electricity consumed in the cathodic process at the alumina electrode, relative to the amount of electricity consumed in the cathode process at the ZnMOF electrode, is about 20% in 1 M NaOH. The same ∼20% is also the difference in the amount of electricity of the anode and cathode processes for the MOF composite in 1 M NaOH. This could be an indirect confirmation of the contribution of hydrogen evolution to the cathode region. Moreover, most likely, hydrogen is released at the initial stages of the process, and only then a parallel process of zinc reduction is implemented. In this case, the latter process also has a change in the nature of the surface, possibly with a phase transformation, judging by the potential shift and a strong decrease in the polarization resistance. If our reasoning is correct, then in the initial sections of the cathode branch of the forward run, we can try to isolate the pure process of hydrogen evolution using the slow charge transfer equation.

Since the overpotential values do not allow using the approximation of a semi-logarithmic dependence for the analysis, a non-linear regression analysis of the initial sections of these curves was carried out according to the full Butler–Volmer equation (Fig. S3†). The approximation was carried out using the OriginPro 2018 software to estimate the degree of closeness of the calculated parameters for the ZnMOF electrode in 1 M and 10 M NaOH electrolytes, as well as for the Al_2_O_3_ electrode in 1 M NaOH. The following equation was used:
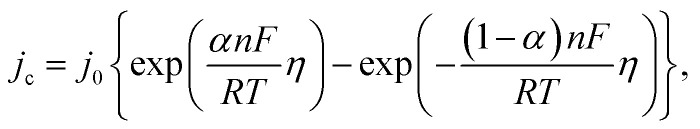
where: *j*_c_ – electrode current density, A m^−2^; *j*_0_ – exchange current density, A m^−2^; *T* – absolute temperature, K; *n* – number of electrons involved in the electrode reaction; *F* – Faraday constant, C mol^−1^; *α* – cathodic charge transfer coefficient, dimensionless; *R* – universal gas constant, J K^−1^ mol^−1^; *η* – activation overpotential, V.

Since zinc reduction is added to hydrogen evolution, the overpotential region was limited by the accuracy of description with a correlation coefficient of at least 0.99. In Fig. S2,† we observe a fairly good approximation of the experimental data by the Butler–Volmer equation with a satisfactory (taking into account the difference in the electrodes) convergence of the calculated parameters obtained in this case (exchange current, transfer coefficient) for all three initial sections for the ZnMOF and Al_2_O_3_ electrodes. However, it should be noted that in 10 M NaOH solution there is some overlap of side processes that cause distortion of the curve. Therefore, we can attribute all these three processes to one, especially if we consider that they are carried out on different electrodes and in different electrolytes. From this, we can draw the main conclusion that hydrogen evolution in each of these cases begins earlier than the second process, most likely, the process of zinc evolution, and the hydrogen and zinc evolution process itself occurs on the carbon surface of the electrodes under study.

In Fig. 5a, we see that the intensity of the peaks falls off as the number of cycles increases. In this case, this may indicate a decrease of the area of the interface, degradation of the material, or the formation of a passivation layer at the electrode–electrolyte interface. At the end of the process, it was found that a white coating of unknown nature had formed on the electrodes. Taking into account that metal–organic compounds formed by ligands based on carboxylates decompose in alkali solutions,^32^ it was assumed that under these conditions, the working material is dissolved in the electrolyte, followed by the formation of a Zn(OH)_2_/ZnO film on the electrode surface. The main impetus for decomposition is the replacement of ligands with hydroxide competitively binding to metal cations of MOF:M^*n*+^ − (RCOO^−^)_*n*_ + *n*OH^−^ = M − (OH)_*n*_ + *n*RCOO^−^

The dissolution of the working material Zn_2_(EDTA)H_2_O probably occurs in several stages with the formation of a zincate. When a critical concentration of zincate is reached at the electrode/electrolyte interface, the formation of a primary passivating ZnO film is initiated. The formation of a passivation layer on the electrode surface prevents the electrochemical reaction from proceeding. A similar phenomenon has already been observed previously.^33–35^

To test this hypothesis, the Zn_2_(EDTA)H_2_O working material was placed in 1 M and 10 M NaOH solutions. It was found that the sample dissolved after 30 minutes confirming its instability in an alkaline environment.

Initially, the electrochemical process in an alkaline electrolyte was presented as a surface reversible reaction Zn^2+^ ⇌ Zn^+^ (ref. 21) in K based electrolyte.

In order to check if there is a significant difference when electrochemical process occurs in the Na based electrolyte instead of K based reported by Patel *et al.*, we studied the working material Zn_2_(EDTA)H_2_O in 0.1 M and 1 M KOH solutions *via* CV. The behaviour in 1 M solutions of NaOH and KOH is very similar with 0.5 V peak shift towards a negative side in Na based electrolyte (Fig. S4a†). This is due to the sodium cation being more acidic with respect to the potassium cation, therefore, the stability of the Na_2_[Zn(OH)_4_] complex appears to be greater than for K_2_[Zn(OH)_4_]. As the KOH concentration lowers, the values of the cathode and anode peak currents decrease (Fig. S4b†). Additionally, a decrease in the concentration of hydroxide ions in the solution leads to a shift in the peak potentials to a more positive region indicating the participation of the hydroxide ion in the electrochemical process. Probably, the dissolution of the working material Zn_2_(EDTA)H_2_O in alkaline electrolytes occurs in several stages with the formation of hydroxozincates, which can subsequently be cycled reversibly according to the following equation:M_2_[Zn(OH)_4_] + 2e = Zn + 2MOH + 2OH^−^

Some of the Zn^2+^ apparently forms ZnO on the surface of the electrode as a decomposition product. This agrees with the precipitate found on the electrode after cycling and the dissolution of MOF in alkaline solutions with the transition of zinc into the electrolyte.

According to published data,^32^ organometallic compounds based on carboxylates are more stable in an acidic medium, since there is no competitive substitution of zinc atoms by protons. However, the reduction potential of zinc is close to the potential of water reduction; therefore, it is also not possible to use acidic media as electrolytes.

**Fig. 6:**
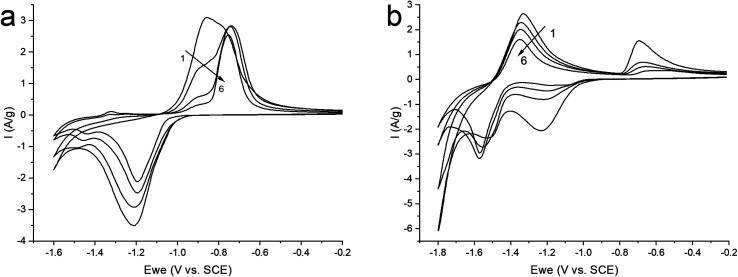
CV curves of ZnMOF in a saturated solution of NaClO_4_ in the potential window: (a) (from −1.6 to −0.2 V); (b) (−1.8 to −0.2 V), at 10 mV s^−1^ sweep rate.

Therefore, in order to stabilize the material, it was decided to switch to a neutral saturated solution of NaClO_4_ where the activity of water is reduced to suppress the competing reaction of H_2_ evolution. Electrochemical tests were carried out in the potential window (from −1.6 to −0.2 V) at a sweep rate of 10 mV s^−1^ in order to study the mechanism of the electrochemical reaction (Fig. 6a). On the graph, we observe cathodic and anodic peaks, which have a rather complex structure, especially the anodic peak, represented by a combination of two merging peaks. Moreover, when cycling, one shoulder of the combined anodic peak significantly decreases, while the second remains practically unchanged. In turn, in the cathodic region, this peak is represented only by a small shoulder at the beginning of the peak. In the more negative region (from −1.4 V), we also see two clearly defined small peaks in the cathode and anode regions.

**Fig. 7:**
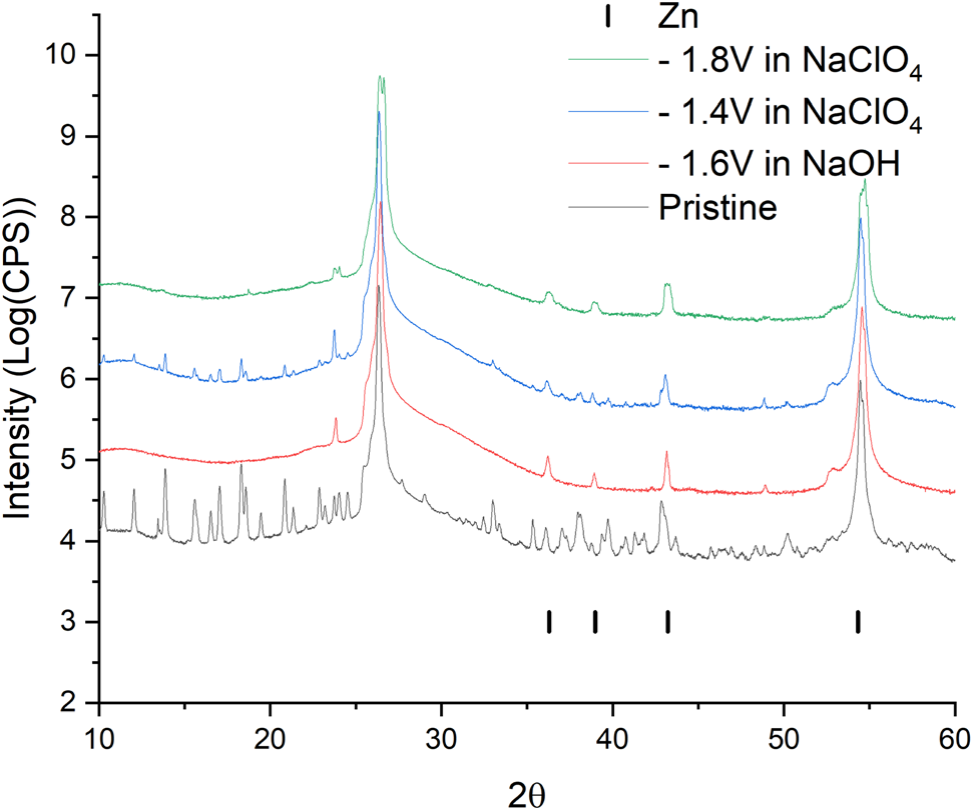
Data from X-ray diffraction analysis of ZnMOF electrode samples after electrochemical study.

For a more intense manifestation of this RedOx process, cyclic voltammetry was carried out in a wider potential window (from −1.8 to −0.2 V). As we can see in Fig. 6b, the intensity of the peaks that appear at a more negative potential has increased significantly. In this case, the potentials of these peaks in the region of hydrogen evolution, as well as the presence of a hysteresis loop in the first cycle, are almost identical both in a saturated NaClO_4_ solution and in an alkaline electrolyte. This may indicate that in a saturated solution of NaClO_4_, the same process of separating metallic zinc from the solution is probably realized. This, in turn, implies that the dissolution of the working material happens already in a neutral environment. However, this raises the question of the nature of the first pair of peaks. Therefore, in order to get a more detailed understanding of the mechanism of RedOx processes occurring in a neutral electrolyte, we studied electrodes after electrochemical cycling in 1 M NaOH and sat. NaClO_4_ using X-ray phase analysis (Fig. 7). X-ray phase analysis of the electrodes showed that in all three cases, after cathodic polarization, upon reduction to −1.8 V (NaClO_4_), to −1.4 (NaClO_4_), and to −1.6 (NaOH), metallic zinc is formed. At the same time, the peaks characteristic of the working material Zn_2_(EDTA)H_2_O are almost absent.

**Fig. 8:**
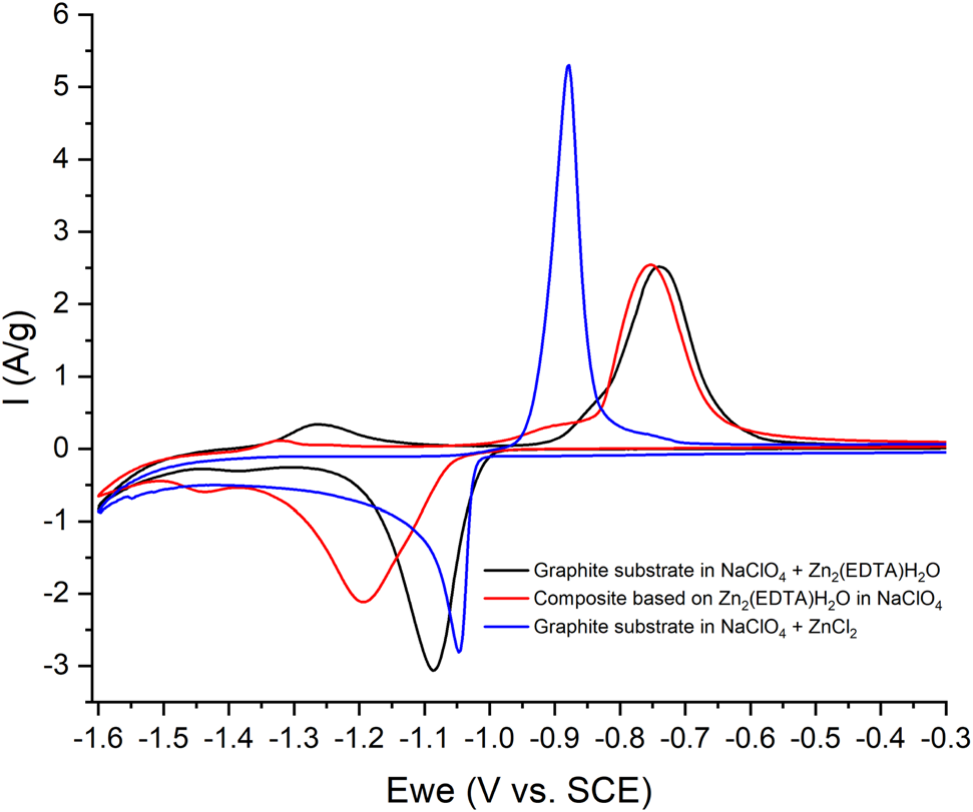
CV-curves of the 3^rd^ cycle: in NaClO_4_ (red) with an electrode based on Zn_2_(EDTA)H_2_O; NaClO_4_ + ZnCl_2_ (blue) and NaClO_4_ + Zn_2_(EDTA)H_2_O (black) with electrodes – graphite foil, at a sweep rate of 10 mV s^−1^.

Thus, we can conclude that both pairs of peaks correspond to the process of separating zinc metal. However, this raises the question of which two forms of zinc are reduced on the carbon surface of the electrode. Considering the possible mechanism of dissolution of a metal–organic compound, we can assume that in solution, most likely, the ZnEDTA complex and the free zinc cation exist in equilibrium. To test this hypothesis, two model electrolyte solutions were prepared. The first solution was obtained by dissolving zinc chloride in a saturated solution of NaClO_4_ (*C*_Zn^2+^_ = 0.044 M), the second was obtained by dissolving the working material Zn_2_(EDTA)H_2_O also in a saturated solution of NaClO_4_ (*C*_Zn^2+^_ = 0.044 M). Electrochemical tests using the obtained electrolytes were carried out in the potential window (from −1.6 to −0.2 V), where graphite foil was used as a working material (Fig. 8). The graph shows a comparison of the CV curves of the Zn_2_(EDTA)H_2_O electrode in a saturated NaClO_4_ solution with the graphite foil electrode in ZnCl_2_ + NaClO_4_ and MOF + NaClO_4_ solutions. As can be seen from the graph, the CV of the Zn_2_(EDTA)H_2_O electrode completely coincides with the CV of the graphite foil electrode in the MOF + NaClO_4_ solution. Some differences in the cathodic region in the form of a small potential shift, as well as a different peak area, are associated with a much higher zinc concentration in the model solution compared to the Zn_2_(EDTA)H_2_O electrode (0.044 M *vs.* 0.0089 M). There is also a complete coincidence of the first peak in the cathodic region in the model solutions ZnCl_2_ + NaClO_4_ and MOF + NaClO_4_. This may be an evidence of the reduction of the free form of zinc at this potential. A significant shift of the anodic peak in the ZnCl_2_ + NaClO_4_ solution is most likely associated with the activation of the anodic process of dissolving metallic zinc chloride with anions.^36^ The observed less intense peaks in the MOF + NaClO_4_ solution at more negative potentials fully correspond to the similar peaks obtained on the Zn_2_(EDTA)H_2_O electrode. In the absence of the EDTA solution (electrolyte ZnCl_2_ + NaClO_4_), the peaks that appear at a more negative potential and probably correspond to complexed zinc are not detected. However, given that the ZnEDTA complex is very strong (*K*_f_ = 10^13^),^37^ the ratio of the reduction peaks of the two forms of zinc seems rather strange, since under such conditions almost no free zinc should be observed in the solution. The only explanation for this contradiction may be the strong dependence of the complex formation constant on the pH of the solution, due to the conjugation of this reaction with the hydrate formation reaction. The possibility to consider the reaction of hydrate formation consists in taking into account the dependence of the complexation constant on the pH of the solution, which for the used MOF + NaClO_4_ solution is 1.86. At a given pH value, EDTA with zinc cation form a protonated complex, the stability of which is quite low (*K*_f_ = 3.16 × 10^2^).^37^ Using the complexation constant under these conditions and the concentration of zinc in solution, the ratio of free and complexed forms of zinc was calculated being only 1 : 3.3. In this case, such a ratio (1 : 3.8) is the ratio of the amount of electricity consumed in the first cathodic process in relation to the amount of electricity consumed in the second cathodic process (Fig. 6b).

The summary of the behaviour of Zn_2_(EDTA)H_2_O in NaOH and NaClO_4_ can be described with the following scheme:
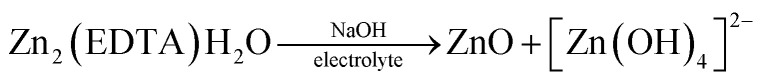

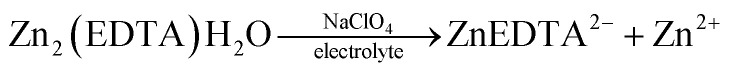


Thus, based on the above results, we can say that the process of cation intercalation/deintercalation into the Zn_2_(EDTA)H_2_O structure in aqueous solutions (both neutral and alkaline) cannot be realized due to its rather high solubility. Therefore, the use of this material as an anode for water-based metal-ion batteries is questionable.

## 4 Conclusions

In this work, we have studied the possibility of using the Zn_2_(EDTA)(H_2_O) MOF composite as an anode material for sodium-ion batteries based on aqueous electrolytes. The structure of the synthesized substance was confirmed by X-ray diffraction analysis with subsequent refinement of the structure by the Rietveld method, as well as by IR spectroscopy. The electrochemical behaviour of this MOF was studied using cyclic voltammetry in alkaline electrolytes 1, 10 M NaOH, as well as in saturated aqueous electrolyte NaClO_4_. It has been established that the studied compound does not give a satisfactory electrochemical response in aqueous electrolytes (both in alkaline and neutral media) due to the strong degradation of the electrode material, which is associated with the high solubility of Zn_2_(EDTA)(H_2_O) in electrolytes.

Cyclic voltammetric studies of Zn_2_(EDTA)(H_2_O) showed the presence of two RedOx processes caused by the release of metallic zinc from an electrolyte solution, where two forms of zinc exist in equilibrium (the ZnEDTA complex and the free zinc cation). The electrochemical activity of this material is manifested in the region of negative potentials (from −1.8 to −0.2 V). Therefore, in the cathodic region, the process of separating metallic zinc is combined with the process of hydrogen reduction, which corresponds to the potentials in this region.

Based on the above, we can conclude that it is not possible to use this material as an anode for water-based batteries. Furthermore, we suggest, that the future studies of the various MOF capabilities for aqueous batteries must be done responsibly and the reports on such studies to be read with care.

## Author contributions

Alena A. Starodubtseva: conceptualisation, data curation, investigation, methodology writing-original draft; Yaroslav S. Zhigalenok: data curation, formal analysis, methodology, writing-original draft; Kairgali M. Maldybaev:investigation; Alina K. Galeyeva: validation, funding acquisition; Ivan A. Trussov: data curation, methodology, supervision, formal analysis, writing – review & editing; and Andrey P. Kurbatov: methodology, validation, writing – review & editing.

## Conflicts of interest

There are no conflicts to declare.

## Supplementary Material

RA-013-D3RA00040K-s001
