# Supplementary material for: On electrochemistry of metal–organic framework Zn2(EDTA)(H2O)
Source: RSC Adv. 2023 Feb 7;13(8):4880–9. doi: 10.1039/d3ra00040k (PMC9903352; doi:10.1039/d3ra00040k)
Supplement: RA-013-D3RA00040K-s001 [file RA-013-D3RA00040K-s001.pdf]

Electronic Supplementary Material (ESI) for RSC Advances.
This journal is © The Royal Society of Chemistry 2023

Supplementary information for: On electrochemistry of metal-organic framework $Zn_2(EDTA)(H_2O)$

A. A. Starodubtseva[1], Y.S. Zhigalenok[1], K. M. Maldybaev[1], A.K. Galeyeva[1], I.A. Trussov[1,2]*, A.P. Kurbatov[1]

[1]*Center of Phisico-Chemical Methods of Research and Analysis, al-Farabi Kazakh National University, Almaty, 010000, Kazakhstan*

[2]*Skoltech Center for Electrochemical Energy Storage, Skolkovo Institute of Science and Technology, Moscow, 121205, Russian Federation*

Table 1S. The list of experiments on ZnMOF synthesis conducted in the work.

Reagents	Synthesis conditions				W, %	Note
	C, M	T, °C	t, h	pH		
$ZnCl_2$<br>$Na_2H_2EDTA \times 2H_2O$	0,085<br>0,085	200	18	3-3.4	0	no solid phase, the presence of a resinious organic phase
$ZnCl_2$<br>$Na_2H_2EDTA \times 2H_2O$	0,085<br>0,085	200	24	3-3.4	0	no solid phase, the presence of a resinious organic phase
$ZnCl_2$<br>$Na_2H_2EDTA \times 2H_2O$	0,085<br>0,085	200	48	3-3.4	34	the presence of a resinious organic phase, particle size of ~27 μm is formed
$ZnCl_2$<br>$Na_2H_2EDTA \times 2H_2O$	0,085<br>0,085	200	72	3-3.4	63	the presence of a resinious organic phase, particle size of ~65 μm is formed
$ZnCl_2$<br>$Na_2H_2EDTA \times 2H_2O$	0,085<br>0,085	200	96	3-3.4	67	the presence of a resinious organic phase, particle size of ~74 μm is formed
$ZnCl_2$<br>$Na_2H_2EDTA \times 2H_2O$	0,085<br>0,085	100	72	3-3.4	0	no solid phase
$ZnCl_2$<br>$Na_2H_2EDTA \times 2H_2O$	0,085<br>0,085	120	72	3-3.4	0	no solid phase
$ZnCl_2$<br>$Na_2H_2EDTA \times 2H_2O$	0,085<br>0,085	160	72	3-3.4	0	no solid phase
$ZnCl_2$<br>$Na_2H_2EDTA \times 2H_2O$	0,085<br>0,085	170	72	3-3.4	60	particle size of ~39 μm is formed, no organic phase
$ZnCl_2$<br>$Na_2H_2EDTA \times 2H_2O$	0,085<br>0,085	180	72	3-3.4	71	particle size of ~42 μm is formed, no organic phase
$ZnCl_2$<br>$Na_2H_2EDTA \times 2H_2O$	0,085<br>0,085	190	72	3-3.4	67	the presence of a resinious organic phase, particle size of ~55 μm is formed
$ZnCl_2$<br>$Na_2H_2EDTA \times 2H_2O$	0,085<br>0,085	210	72	3-3.4	0	no solid phase, the presence of a resinious organic phase
$ZnCl_2$<br>$Na_2H_2EDTA \times 2H_2O$	0,085<br>0,085	220	72	3-3.4	0	no solid phase, the presence of a resinious organic phase
$ZnCl_2$<br>$Na_2H_2EDTA \times 2H_2O$	0,085<br>0,085	180	72	1.6	0	no solid phase, not used KOH

$ZnCl_2$<br>$Na_2H_2EDTA \times 2H_2O$	0,085<br>0,085	180	72	2.5	0	no solid phase, before the synthesis, there is no complete dissolution of $Na_2H_2EDTA \times 2H_2O$
$ZnCl_2$<br>$Na_2H_2EDTA \times 2H_2O$	0,085<br>0,085	180	72	5.4	0	no solid phase, before the start of synthesis, the appearance of blue flakes $Zn(OH)_2$
$ZnCl_2$<br>$Na_2H_2EDTA \times 2H_2O$	0,17<br>0,17	180	72	3-3.4	73	particle size of ~62 μm is formed, no organic phase
$ZnCl_2$<br>$Na_2H_2EDTA \times 2H_2O$	0,34<br>0,34	180	72	3-3.4	70	particle size of ~75 μm is formed, the presence of a resinious organic phase

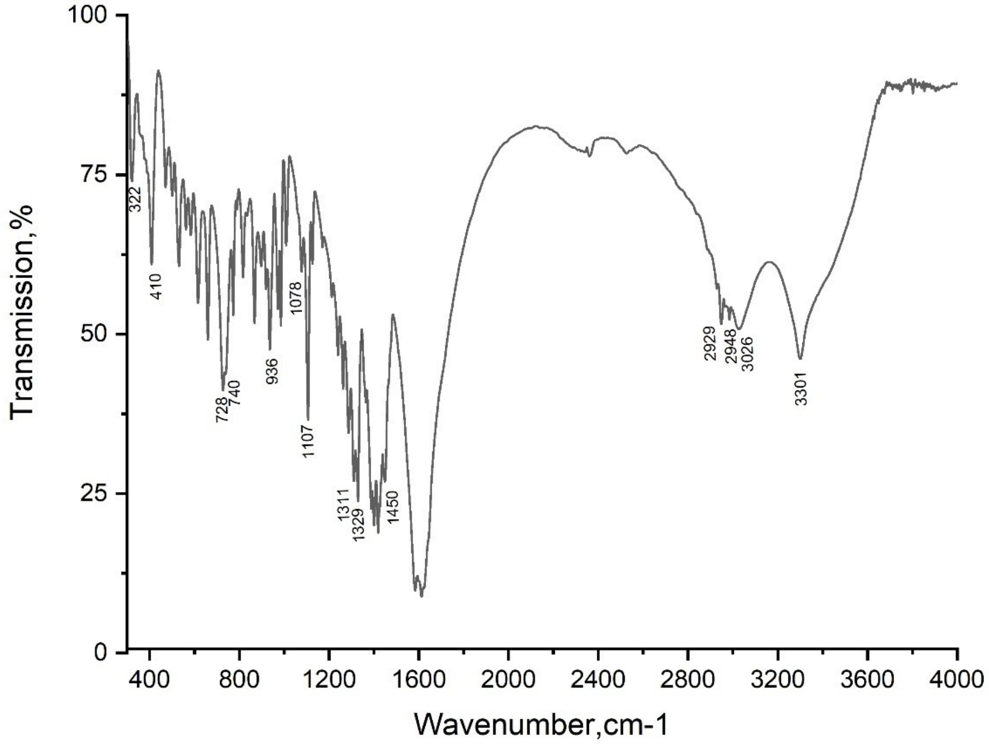

Figure S1- Infrared spectrum of $Zn_2(EDTA)H_2O$

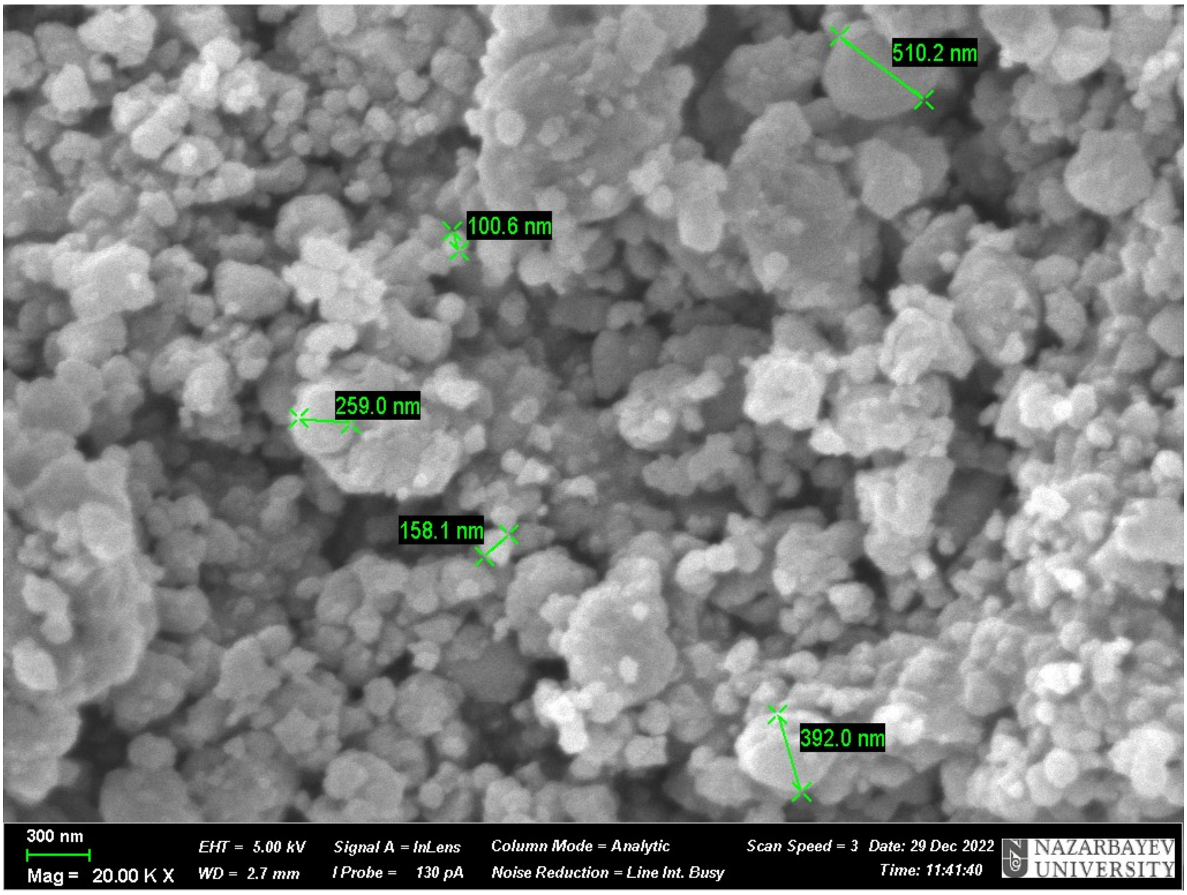

Figure S2 - SEM images of $Zn_2(EDTA)H_2O$ sample after ball-milling with sizes.

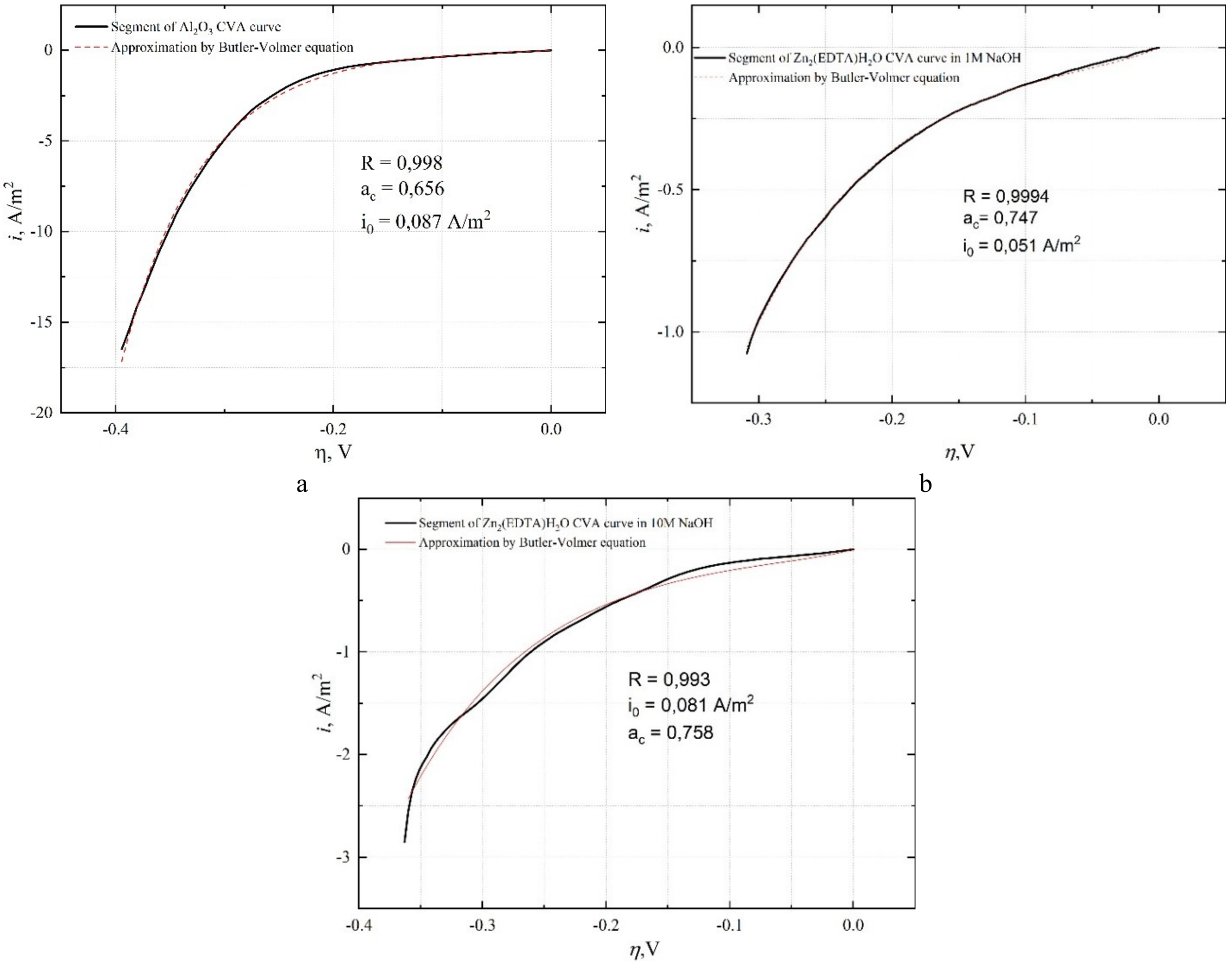

c

Figure S3 - sections of cyclic voltammograms interpolated by the Butler-Volmer equation, a) an electrode based on Al_2O_3 in 1M NaOH; b) an electrode based on $Zn_2(EDTA)H_2O$ in 1M NaOH; c) electrode based on $Zn_2(EDTA)H_2O$ in 10M NaOH

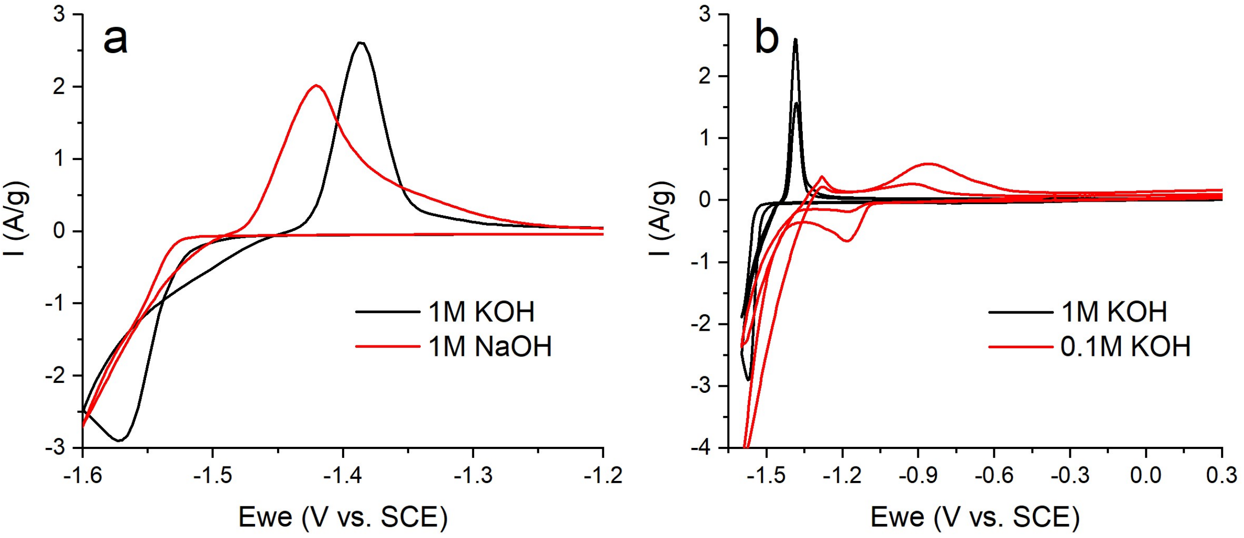

Figure S4 – CV curves of $Zn_2(EDTA)H_2O$ sample at 10 mV/s in a) 1M KOH and NaOH solution; b) 1M and 0.1 M KOH.
